# Tumor-Associated Macrophage Subsets: Shaping Polarization and Targeting

**DOI:** 10.3390/ijms24087493

**Published:** 2023-04-19

**Authors:** Qindong Zhang, Mouldy Sioud

**Affiliations:** 1 Division of Cancer Medicine, Department of Cancer Immunology, Oslo University Hospital, University of Oslo, Ullernchausseen 70, 0379 Oslo, Norway; qindong.zhang@rr-research.no; 2 Department of Pharmacy, Faculty of Mathematics and Natural Sciences, University of Oslo, Blindern, P.O. Box 1068, 0316 Oslo, Norway

**Keywords:** tumor-associated macrophages, tumor microenvironment, polarization, cell signaling, targeted therapy, solid cancers

## Abstract

The tumor microenvironment (TME) is a critical regulator of tumor growth, progression, and metastasis. Among the innate immune cells recruited to the tumor site, macrophages are the most abundant cell population and are present at all stages of tumor progression. They undergo M1/M2 polarization in response to signals derived from TME. M1 macrophages suppress tumor growth, while their M2 counterparts exert pro-tumoral effects by promoting tumor growth, angiogenesis, metastasis, and resistance to current therapies. Several subsets of the M2 phenotype have been observed, often denoted as M2a, M2b, M2c, and M2d. These are induced by different stimuli and differ in phenotypes as well as functions. In this review, we discuss the key features of each M2 subset, their implications in cancers, and highlight the strategies that are being developed to harness TAMs for cancer treatment.

## 1. Introduction

The tumor microenvironment (TME) plays a critical role in the aggressive behavior of human solid malignancies. Among the cell types associated with the TME, tumor-associated macrophages (TAMs) and their precursors account for the largest fraction of the myeloid infiltrate in the majority of solid tumors [1]. Most often, high levels of TAMs in the TME are associated with cancer progression, metastatic spread, poor treatment efficacy, and shorter survival in several cancer types such as lung cancer [2,3], breast cancer [4,5,6,7], hepatocellular carcinoma (HCC) [8], gastric cancer [9], and melanoma [10]. It is assumed that circulating monocytes serve as a primary source for TAMs that shape the TME. Moreover, tissue resident macrophages, originating from progenitors of the yolk sac, can also polarize into TAMs [11]. Similarly, monocytic myeloid-derived suppressor cells (M-MDSCs) can be recruited into tumors and differentiate into TAMs [12]. Among the TAM precursors, monocytes are important mediators in the crosstalk between monocytes/macrophages and tumor cells. Compared to normal tissues, tumors release various attractants (e.g., M-CSF, CCL2 and CCL5) to recruit monocytes [11,13]. The infiltrating monocytes, M-MDSCs, and tissue resident macrophages are then educated by the TME and differentiate into TAMs, which in turn produce various types of tumor supportive factors (e.g., EGF and TGF-β) to promote tumor growth, angiogenesis, and metastasis [13,14]. Additionally, TAMs utilize several strategies to help tumor cells evade immune surveillance and elimination by establishing an immunosuppressive microenvironment. For instance, TAMs can facilitate the recruitment of immunosuppressive cells such as Treg and Th2 cells by releasing chemokines including CCL2 and CCL20 [11,13,15,16].

After entering peripheral tissues, the pattern of macrophage activation depends on the signals received from the local microenvironment, which accounts for their functional plasticity and heterogeneity. An elevated degree of heterogeneity in TAM populations has been described across different cancer patients [8]. Once activated, naïve macrophages (M0) can be polarized into two main subsets: M1 and M2. The M1 phenotype is considered pro-inflammatory and is correlated with the production of pro-inflammatory cytokines including TNF, IFN-γ, and IL-12. In contrast, the M2 phenotype is anti-inflammatory and is correlated with the release of anti-inflammatory cytokines such as IL-10, TGF-β, and IL-6 [3,6,7,17,18,19,20,21].

Established tumors often display abundant numbers of TAMs and their presence is associated with increased tumor progression and invasion [13,14,22]. They can account up to 50% of the tumor mass [13,22]. Of note, the TME is known to predominantly polarize TAMs toward the M2 phenotype [8]. Most commonly, in contrast to M1, M2 macrophages are negatively associated with longer survival time and positive clinical outcome in multiple types of cancers including small cell lung cancer, colorectal cancer, ovarian cancer, and breast cancer [14,22,23]. Hence, TAMs usually refer to M2 macrophages. Malignant cells can secrete M2-like cytokines such as IL-10, CCL2, CXC12, VEGF, and PDGF to recruit more monocytes and M0 macrophages to the tumor sites. The location of TAMs within the TME was found to affect their ability to support versus restrain tumor progression [1]. In tumors, endothelial cells and macrophages also interact with each other to favor tumor progression. TAMs favor angiogenesis and shift the endothelial cell phenotype toward one promoting cancer cell intravasation and extravasation [24,25,26,27,28]. Moreover, endothelial cells are significantly involved in the regulation of monocyte/macrophage infiltration in the tumors, and infiltrated monocytes/macrophages enhance cancer cell extravasation, a crucial step in cancer metastasis. This knowledge can be harnessed for the design of inhibitors that will interfere with the interaction between TAMs and activated endothelium [24,28]. Here, we present the major factors that contribute to the formation of these specific M2 subsets, their implications in tumor progression, and discuss various therapeutic strategies to harness TAMs for cancer treatment.

## 2. M2-like Macrophages

The concept of classically (M1) and alternatively (M2) activated macrophages was originally proposed by Mills et al. [29]. This classification is based on the metabolism of L-arginine in response to Th1 (IFN-γ and/or LPS) and Th2 (IL-4/IL-13) stimulation. IFN-γ and/or LPS-induced M1 macrophages upregulate the expression of inducible nitric oxide synthase (iNOS), which metabolizes L-arginine to L-citrulline and nitric oxide. In contrast, IL-4/IL-13 stimulation promotes the M2 phenotype that is characterized by the upregulated expression of arginase 1 (Arg1), which metabolizes L-arginine to various tumor-supporting factors (e.g., L-ornithine and polyamines) [29,30,31]. Although the M1 and M2 paradigm has provided a useful guide for studying macrophage polarization and function, it is now considered as an oversimplification. Additionally, this classification does not consider the sources and tissue microenvironment. Over the years, macrophages that share similar properties with M2 but are primed by stimuli other than IL-4/IL-13 have been described [30,31]. However, they do not fit well into the criteria for either M1 or M2 macrophages. Based on the stimuli they receive as well as the resultant phenotypes and functions, these M2-like macrophages are further divided into four subsets, named M2a, M2b, M2c, and M2d. Although they exhibit different phenotypic markers, gene expression profiles, cytokine profiles, and functional activities, all share an IL-10^high^ and IL-12^low^ cytokine profile [11,32,33,34,35]. Below, the representative features of each M2 subset and their implications in cancers are discussed.

### 2.1. M2a Macrophages

The M2a macrophages historically represent the most widely studied M2 subset. These macrophages were first described in 1992 by a study demonstrating that IL-4 stimulated murine peritoneal macrophages had enhanced surface expression of CD206 with increased functional activity [36]. Apart from IL-4, IL-13 is another cytokine that can polarize macrophages toward the M2a phenotype, which is characterized by the high expression of cell surface markers CD206, CD209, and Dectin-1. The expression levels of CD14, CD163, and CD80/86 vary from low to medium. M2a cells produce IL-10, CCL17, CCL18, CCL22, and the amino-acid catabolizing enzyme Arg1 [3,6,7,17,18,19,20,21]. These phenotypic markers and intracellular factors define the functional activities of M2a.

As above-mentioned, M2a macrophages express certain pattern recognition receptors (PRRs) such as CD206, CD209, and Dectin-1, thus facilitating the sensing and elimination of invading bacteria, fungi, and parasites. The binding of pathogen-derived carbohydrates to PRRs triggers scavenging activities and activates downstream signaling cascades, leading to IL-10 production [37,38]. Secreted IL-10 inhibits pro-inflammatory IL-12 production as well as the expression of co-stimulatory molecules CD80/86, which in turn renders M2a macrophages poor inducers of T cell activation and proliferation [21]. Tissue remodeling is another prominent feature of M2a. In response to tissue damage, IL-4 is released and induces macrophages to differentiate toward M2a, resulting in the production of various cellular products that play major roles in matrix reorganization. For instance, L-ornithine, one of the metabolites generated by Arg1, is a precursor of collagens and polyamines, which are major components of the extracellular matrix (ECM). Certain chitinase-like substances produced by M2a also function in matrix reorganization [34]. It has been reported that M2 but not M1-polarized macrophages expressed high levels of fibronectin, which plays an important role in tissue repair and cell motility [39,40,41]. Hence, fibronection has been proposed as a potential biomarker for M2 macrophages [39]. Fibronectin also induced M2 polarization. Indeed, Zhou et al. found that fibronectin-1 secreted from human head and neck squamous cell carcinoma, co-cultured with THP-1 macrophages, enhanced M2 polarization [42]. Fibronectin also supports tumor progression by promoting tumor cell proliferation, invasion, and migration [42,43,44]. Considering its detrimental roles in cancers, fibronectin is now recognized as a potential target for cancer therapy [44,45].

**Figure 1:**
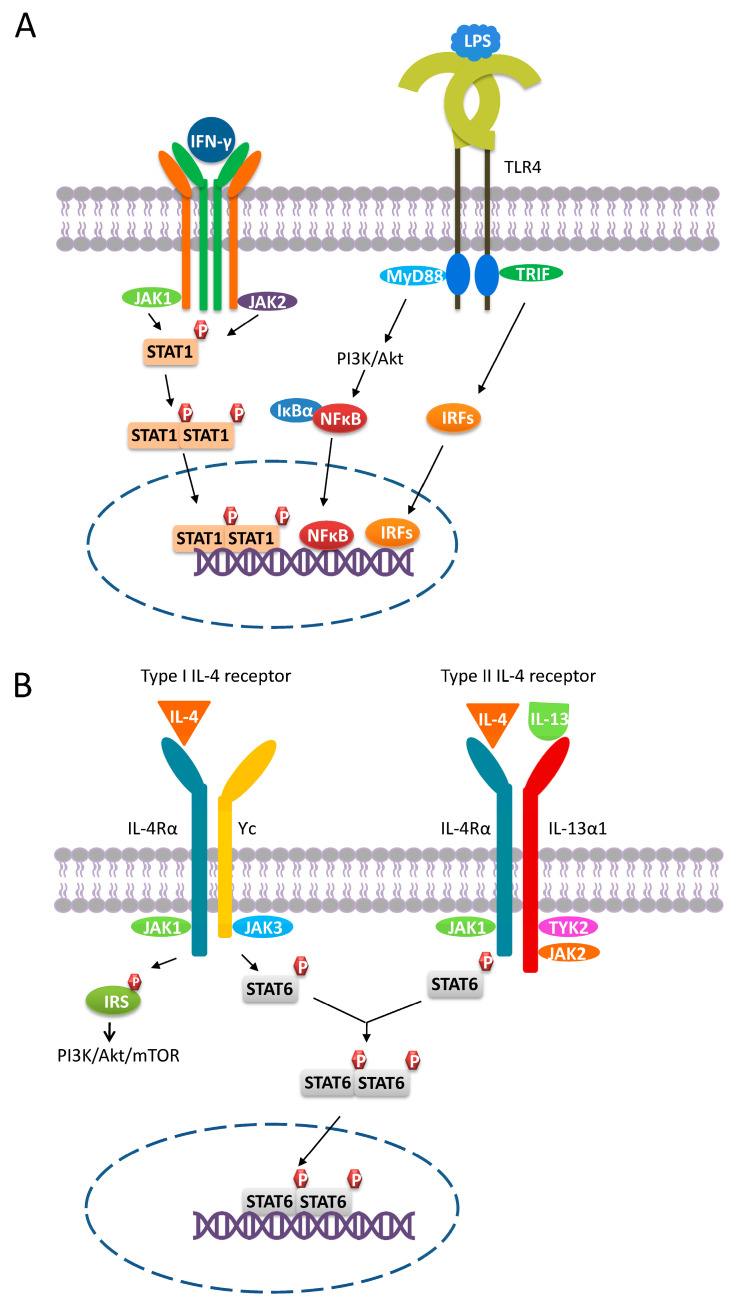
M1 and M2a polarization. (**A**) M1 macrophages polarize through signaling pathways mediated by STAT1, PI3K/Akt, NFκB, and IRFs. Binding of IFN-γ to its receptor leads to the recruitment of JAK1 and JAK2, inducing the phosphorylation of STAT1, which then dimerizes and translocates into the nucleus. In response to the LPS-TLR4 engagement, PI3K/Akt-, NFκB-, and IRFs-mediated signaling are triggered. (**B**) M2a macrophages polarize through the PI3K/Akt- and STAT6-mediated signaling pathways. Macrophages express both type I and type II IL-4 receptors. The engagement of the type I/II IL-4 receptor results in the phosphorylation and subsequent dimerization of STAT3. Once activated, STAT3 dimers translocate into the nucleus and trigger corresponding gene expression. In contrast, IRSs can only be activated by the type I IL-4 receptor and do not translocate to the nucleus. Instead, activated IRSs can induce signaling pathways such as PI3K/Akt-mediated signaling.

IL-4 and IL-13 are produced by several cell types including eosinophils, basophils, mast cells, NKT cells, ILC2 cells, macrophages, Th2 cells, and tumor cells [3,7,37,46,47]. Notably, there are two types of IL-4 receptors (Figure 1A,B). For signaling through the type I receptor, IL-4 first binds to the IL-4Rα chain with high affinity, forming an IL-4/IL-4Rα complex, which then binds to a secondary receptor Ƴc chain. The IL-4/IL-4Rα/Ƴc complex is a functional receptor that enables the transmission of extracellular signals into the intracellular environment. For signaling through the type II receptor, the formed IL-4/IL-4Rα complex assembles with the IL-13Rα1 chain, which is the receptor for IL-13 to form a final functional receptor. Since myeloid cells express both types of IL-4 receptors, the activation of these receptors by either IL-4 or IL-13 would promote macrophage polarization toward M2a [37,46]. Of note, myeloid cells may lose functional IL-4 receptors during transformation. For instance, THP-1 is a human monocytic leukemia cell line that lacks the type I IL-4 receptor [48]. Therefore, THP-1 is not an ideal in vitro model to acquire M2a by stimulation [49,50,51]. The engagement of the type I/II IL-4 receptor induces conformational changes in its intracellular domains, facilitating the activation of downstream signaling molecules including signal transducer and activator of transcription 6 (STAT6) and insulin receptor substrate (IRS) by Janus kinase (JAK). IL-4Rα associates with JAK1, and the Ƴc chain interacts with JAK3, whereas IL-13Rα1 associates with JAK2 and TYK2. Activation of JAKs leads to the phosphorylation of tyrosine residues located on the cytoplasmic domains of the IL-4 receptors, providing docking sites for STAT6 and IRSs, which are then phosphorylated by JAKs to become fully activated. After phosphorylation, activated STAT6 molecules dimerize and translocate into the nucleus where they bind to the promoters of IL-4/IL-13-responsive genes to trigger gene expression. Conversely, IRSs do not translocate into the nucleus, but instead activate various signaling pathways such as the PI3K/Akt/mTOR pathway [37,46]. It has been shown that PI3K/Akt-mediated signaling can enhance the IL-4-driven polarization and proliferation of murine M2 macrophages in vivo [52]. Furthermore, the engagement of the type I IL-4 receptor by IL-4 leads to the activation of both STAT6 and IRS. In contrast, the activation of the type II IL-4 receptor only effectively activates STAT6, whereas IRS is only weakly activated [48]. 

#### Pro-Tumoral Effects of M2a Macrophages

M2a macrophages have been found to promote tumor progression in human. For example, VEGF and CCL18 secreted by M2a macrophages synergistically induce angiogenesis and promote tumor cell migration and invasion in breast cancer [6,7]. During blood vessel formation, both VEGF and CCL18 can promote the mobility of endothelial cells. Nevertheless, only VEGF stimulates endothelial cell proliferation, whereas CCL18 barely has this function [6]. Consistently, M2a macrophages have been found to promote lung cancer growth and invasion in a co-culture system [2]. Furthermore, Fu et al. reported that M2a promoted cancer progression through the IL-4/STAT6 signaling pathway in lung cancer. They also showed that tumor cells relied on macrophages with functional STAT6 to promote tumor cell proliferation, as STAT6 deficient macrophages resulted in a reduced tumor size. This can be explained by the ability of tumor cells to hijack M2a cells to support their growth via the IL-4/STAT6-mediated pathway. IL-4 released from both tumor cells and M2a further promoted more macrophages to polarize toward M2a, which in turn produced more IL-4, thereby forming a positive feedback loop [3]. To conclude, tumor-released factors along with IL-4/IL-13-mediated cell signaling pathways provide M2a macrophages with the ability to promote tumor progression.

### 2.2. M2b Macrophages

**Figure 2:**
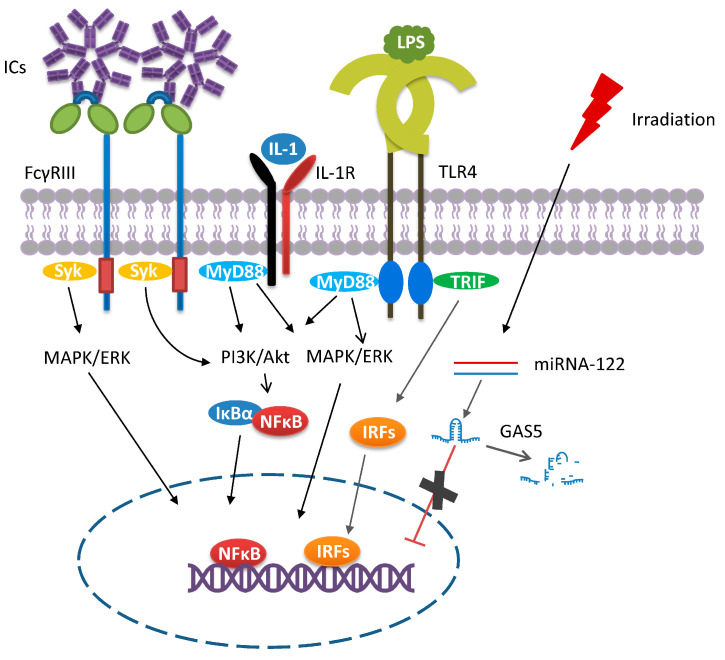
M2b polarization. The macrophage polarization toward the M2b phenotype needs two classical stimuli (ICs and LPS/IL-1). The crosslinking of FcγR by ICs triggers the activation of PI3K-, MAPK/ERK-, and NF-κB-mediated signaling pathways. To fully polarize, the binding of LPS or IL-1 to their respective receptors is also required, which then induces the activation of NFκB, PI3K/Akt, IRFs, and MAPKs. In addition, stimuli (e.g., irradiation, microRNA 122) other than the classical inducers have been described. The presence of long non-coding RNA GAS5 negatively correlates with M2b polarization. In response to irradiation exposure, the induced miRNA-122 expression leads to the degradation of GAS5, thereby favoring M2b polarization.

In 2002, Charles F. et al. found that the addition of TLR agonists (LPS) and immune complexes (ICs, antibody/antigen complexes) could convert macrophage phenotype from M1 to M2 by downregulating IL-12 production and upregulating IL-10. This novel M2 subset was later termed M2b, and thus, LPS plus ICs are now considered as the classical inducers of M2b [53,54]. This macrophage subset has several characteristics that make it distinct from the other M2 subsets. First, the crosslinking of the Fcγ receptor (FcγR) on M2b macrophages induces high levels of anti-inflammatory IL-10 and low levels of IL-12 [17,21,53,54,55,56]. Second, M2b macrophages can bias Th1-cell responses toward Th2-cell responses, predominantly through IL-4 secretion [34,53,54]. Hence, M2b macrophages are also named regulatory macrophages. Third, the polarization toward M2b macrophages require two stimuli, leading to the activation of several signaling events involving NFκB, PI3K/Akt, IRFs, and MAPKs [34,53,57,58] (Figure 2). Although the underlying mechanisms leading to cell polarization remain unclear, they resemble those induced by IC vaccines. In vitro preformed IgG/antigen complexes as well as those formed following antibody therapy in vivo are multifaceted immune regulators. A landmark turning point for IC’s immune regulatory functions was the discovery of FcγRs [59]. Some of these receptors transmit activating signals via an immunoreceptor tyrosine-based activation motif (ITAM) on the associated common γ chain. Upon IC binding to these receptors, the ITAM/syk interaction is the primary signaling axis in phagocyte activation, leading to the activation of the PI3K, PLC, MAPK, and NF-κB signaling pathways [60]. In the case of macrophage polarization, the binding of the ICs to one of the Fcγ receptors will not fully induce macrophage polarization on its own. The cell needs to receive a second signal such as the binding of LPS and IL-1 to their respective receptors (Figure 2). It is only when these two stimuli coexist that the M1 phenotype can reprogram to M2b. Of note, M2b cells, but not other M2 subsets, produce high levels of CCL1 [19,53,54,57,61]. The released CCL1 is essential in maintaining the M2b properties as its inhibition led to the conversion of M2b to M0 or M1 macrophages [8,54,56,62].

Other factors are proposed as good markers in discriminating M2b, but their use still remains controversial. (1) LIGHT (also known as CD258 or TNFSF14) is a secreted protein and can compete with the herpes simplex virus for cell-binding, thus inhibiting viral entry into the cells. LIGHT has been shown to be exclusively upregulated in M2b macrophages in comparison to other macrophage subsets such as M1 and M2a [21,56]. In contrast, Wang et al. showed that M2b derived from human blood monocytes primed by LPS plus IL-1β only expressed very low levels of LIGHT [18]. Sphingosine kinase 1 (SPHK1) was also found to be solely upregulated in M2b [21,56], supporting its use as a marker. However, the use of SPHK1 is controversial as the expression of SPHK1 is not restricted to M2b. For example, SPHK1 was found to be expressed at high levels in the M1 compared to M2 macrophages. In addition, its expression was observed in the M2c subset [54]. CD86 as well as TNF-α were also claimed to be good markers to discriminate M2b from the other M2 subsets. However, CD86 was also reported to be expressed at higher levels in human M2a in comparison to other M2 subsets [17,61]. The L-arginine metabolism pathway has also been proposed as a way to discriminate between different M2 subsets [11]. Murine M2b macrophages have been reported to produce high levels of NO, a metabolite of the iNOS pathway. In contrast, Ito et al. found neither iNOS nor Arg1 RNA expression in ICs and LPS primed murine macrophages [19]. In line with this finding, TAMs isolated from intermediate-stage hepatocellular carcinoma (HCC) patients were identified as M2b macrophages, but barely any iNOS mRNA expression was observed [8]. 

In addition to the classical inducers, other stimuli of M2b macrophages have been identified [54,58]. For example, Chen et al. reported that activated lymphocyte derived DNA (ALD-DNA), which functions as an inducer of lupus nephritis, can polarize macrophages toward M2b [58]. This is because ALD-DNA can be recognized by intracellular TLRs and initiates downstream signaling. By complexing with anti-DNA antibodies, ALD-DNA activates the FcγR mediated signaling pathways as part of the ICs [54]. Radiation and microRNA-122 (miRNA-122) are other identified inducers. Growth arrest specific 5 (GAS5) is a long non-coding RNA and it has been validated as a silencer of the gene encoding CCL1 [63], which is critical in maintaining M2b properties, and thus its overexpression is negatively correlated with M2b polarization [19]. The induction of miRNA-122 expression in murine bone marrow-derived macrophages in response to radiation led to the degradation of GAS5 RNA and macrophage polarization to the M2b subset (Figure 2). Interestingly, the downregulated GAS5 expression level was only observed in M2b macrophages, but not for other macrophage subsets [19], indicating that GAS5 may be a good marker for discriminating M2 subsets.

#### Pro-Tumoral Effects of M2b Macrophages

Several lines of evidence demonstrate that M2b macrophages are present in the TME and play a role in promoting tumor progression. In HCC, only a few M2b macrophages are detected at an early stage. However, as the tumor progressed, the number of M2b cells increased and gradually took over the M1 cell population [8]. This is highly correlated with the CCL1/CCR8 axis. Indeed, the high levels of CCL1 secreted by M2b macrophages attract CCR8 expressing Th2 and Treg cells, promoting an immunosuppressive microenvironment [57]. Additionally, the engagement of CCL1 with CCR8 expressed on tumor cells (e.g., melanoma, bladder cancer) promoted tumor cell proliferation, migration, and metastasis [63,64]. Liu et al. described the presence of M2b macrophages in the TME of bevacizumab resistant triple-negative breast cancer. This was explained by the interaction of the antibody bevacizumab with FcγR along with the activation of the TLR4-mediated pathway by TLR agonists present in the TME, leading to the polarization of TAMs toward the M2b phenotype. However, it is unclear as to which TLR agonist may have contributed to the M2b polarization. The TLR4 agonist, high mobility group box 1 (HMGB1), might be involved because it was largely elevated in bevacizumab-resistant breast cancer [5]. Moreover, M2b macrophages were able to promote breast cancer cell migration and metastasis via TNF-α, as the depletion of TNF-α in M2b culture medium reduced the migration of tumor cells in a co-culture system [5]. Furthermore, indoleamine 2,3-dioxygenease (IDO) expression was upregulated by TNF-α in M2b [5]. In human HCC, infiltrating B cells cooperate with the TME to induce the generation of M2b cells marked by increased IL-10 and CCL1 production. IgG secreted from infiltrating B cells formed ICs with tumor derived antigens, which in turn crosslinked Fcγ receptors displayed on macrophages localized within the TME. Similar to breast cancer, the TLR4 signaling pathway was found to be involved in the polarization of M2b macrophages since its blockade impaired the upregulation of M2b markers in HCC [65].

Additionally, M2b macrophage secreted factors (e.g., IL-10, IL-6, IL-1) contribute to the pro-tumoral effects. For instance, IL-10 has not only been proven to be a potent anti-inflammatory cytokine that can inhibit the production and functional activities of various pro-inflammatory cytokines [34], but also favors the differentiation of naïve T cells into Treg cells [66]. M2b macrophages also produce IL-6 in response to ICs or other stimuli that induces the activation of Th2 cells, inducing the activation of Th2 cells, which promote tumor progression [54]. M2 macrophage-derived exosomal microRNA-155-5p was found to be associated with increased IL-6 expression. This microRNA promotes immune escape and cancer cell metastasis [67].

### 2.3. M2c Macrophages

M2c are macrophages stimulated by IL-10, TGF-β, or glucocorticoids. In comparison to other macrophage subsets, M2c macrophages express high levels of the cell surface markers CD163, Mer tyrosine kinase (MerTK), and Tie2 as well as low to medium levels of CD14, CD86, CD16, and CD206 [17,18,20,68]. The expression levels of the surface markers described above depend on the culture conditions, especially the concentrations and type of stimuli (IL-10, TGF-β, or glucocorticoids), culture medium components (with or without serum), cell origins (murine or human), and cell types (primary cells or cell lines). Usually, M2c macrophages are characterized by the secretion of the pro-inflammatory cytokines IL-10 and TGF-β as well as the chemokines CCL16, CCL18, and CXCL13 [19,69,70]. In terms of L-arginine metabolism, M2c macrophages share an identical metabolic state with M2a and produce Arg1 [19,69].

**Figure 3:**
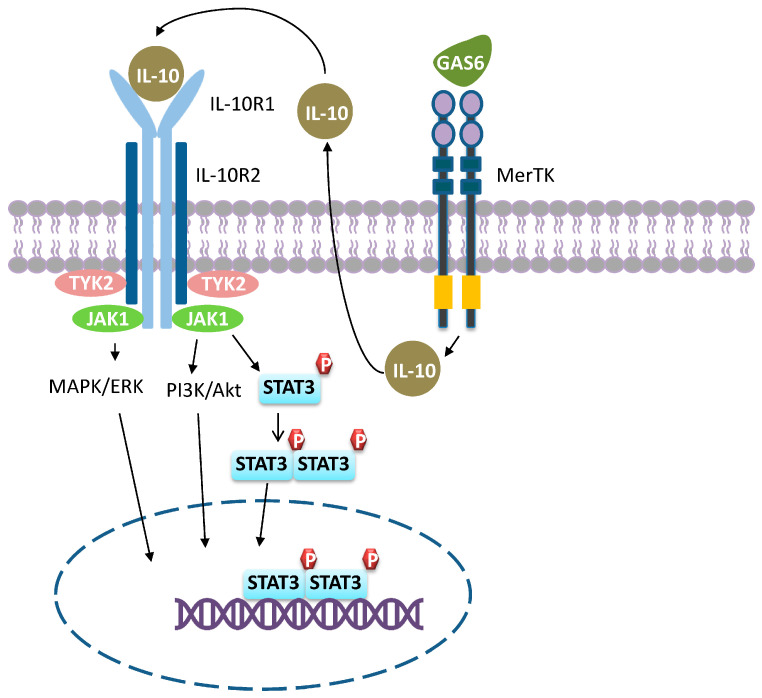
M2c polarization. M2c macrophages polarize through signaling pathways mediated by STAT3, MAPK/ERK, and PI3K/Akt. Engagement of GAS6 produced by M2c with MerTK induces IL-10 production, which in turn activates M2c, resulting in a positive loop that favors M2c polarization.

IL-10 is produced by virtually all types of leukocytes such as macrophages, T, and B cells [71]. The IL-10 receptor (IL-10R) is a tetramer consisting of two IL-10R1 and two IL-10R2 subunits. As shown in Figure 3, IL-10R1 functions as a ligand binding site and associates with JAK1, whereas IL-10R2 acts as a signal transduction subunit and interacts with TYK2. Binding of IL-10 to IL-10R induces receptor auto-phosphorylation and subsequent recruitment of the downstream transcription factor STAT3. Nuclear translocation of dimerized STAT3 activates the transcription of anti-inflammatory genes and downregulates the expression of pro-inflammatory cytokines [31,72,73,74,75]. IL-10 prevents monocytes from differentiating to dendritic cells (DCs) and skews macrophage polarization [76]. Upon IL-10 exposure, macrophages upregulate the expression of an increasing number of genes associated with anti-inflammatory activities in a time-dependent manner. In the meantime, the ability to respond to M1 stimuli (LPS and IFN-Ƴ) is gradually lost through the inhibition of STAT1 and NF-κB, resulting in suppressed M1 activation (Figure 1A) [77,78]. As a consequence, M2c macrophages are also called acquired deactivation macrophages because they lose their ability for M1 polarization [74]. Of note, it is possible to revert M2c macrophages to M1 if STAT3-mediated signaling is blocked. Anderson et al. showed that human M2c macrophages were converted to M1 and exhibited an increased expression of pro-inflammatory cytokines upon delivering corosolic acid-loaded liposomes into the cells through CD163-mediated endocytosis [79]. Corosolic acid is a STAT3 inhibitor and the delivery of corosolic acid-loaded liposomes into macrophages prevents M2c polarization while favoring M1 polarization. Apart from STAT3-mediated signaling, lines of evidence suggest that IL-10 also signals through the PI3K/Akt and MAPK pathways [75]. Signaling through these pathways results in the activation of a series of genes associated with anti-inflammation, matrix remodeling, angiogenesis, and phagocytosis [80].

It should be noted that immunoregulation is a prominent feature of M2c macrophages. To regulate immune responses, M2c suppresses M1-favored inflammation though several mechanisms. First, they deprive macrophages of their ability to polarize into M1 and produce pro-inflammatory cytokines, as described above. Second, they capture and sequester inflammatory chemokines. The expression of certain inflammatory chemokine receptors such as CCR2 and CCR5 are upregulated in IL-10-primed M2c macrophages. Interestingly, these inflammatory chemokine receptors displayed on the cell surface function as decoy receptors as they cannot transmit signals. Instead, they capture inflammatory chemokines and initiate scavenging events [69]. Third, M2c cells are efficient in eliminating apoptotic cells via MerTK-mediated phagocytosis [20]. Clearance of apoptotic cells not only leads to the release of anti-inflammatory cytokines IL-10 and TGF-β from macrophages, but substantial evidence suggests that the apoptotic cells themselves can also trigger anti-inflammation responses [20,81]. Moreover, M2c macrophages produce GAS6, which is the ligand for MerTK (Figure 3). Engagement of GAS6 with MerTK amplifies IL-10 production, which in turn constitutes a positive loop in M2c macrophages, where the resultant anti-inflammation responses can be sustained [20]. Since M2c macrophages act as a rich source of anti-inflammatory cytokines, the presence of M2c results in anti-inflammatory cytokine release. Tissue remodeling is another functional property of M2c macrophages. They degrade the ECM by secreting high levels of MMP7, MMP8, MMP9, and TIMP1 [80].

#### Pro-Tumoral Effects of M2c Macrophages

Emerging evidence implicates M2c macrophages in supporting tumor progression. For example, in patients with breast, it has been demonstrated that the percentage of circulating M2c macrophages is correlated with disease severity [4]. In line with this, Yuan et al. demonstrated that M2c macrophages promoted xenograft lung tumor growth in vivo and induced tumor cell invasion in vitro [2]. Kim et al. provided indirect evidence showing that M2c promoted tumor growth in mice bearing melanoma or lymphoma. Pellino-1 protein was originally identified as a ubiquitin ligase that plays a role in regulating TLR signaling by acting as a scaffolding protein. In their study, they found that pellino-1 regulates STAT3 activation via enhancing STAT1 signaling, thereby damping IL-10-induced M2c macrophage polarization while favoring M1 polarization in mouse BMDMs. Consistent with this, they also showed that pellino-1-deficient mice harboring a significantly reduced M1/M2 ratio and increased tumor size [10]. Overall, these observations suggest that M2c macrophages favor tumor growth while pellino-1 inhibits tumor growth by suppressing M2c polarization. Additionally, M2c may support tumor growth by inducing angiogenesis as the M2c macrophages facilitated endothelial cell mobility and tube formation in vitro and in vivo [82,83].

### 2.4. M2d Macrophages

M2d macrophages were identified in 2007 in the ascites of ovarian patients. It was found that IL-6 and leukemia inhibitory factor (LIF) were present at high levels in the ascites, with only moderate levels of M-CSF and undetectable levels of cytokines, which can skew macrophages toward other macrophage subsets such as M1, M2a, M2b, and M2c. The study demonstrated that IL-6 and LIF polarized macrophages to a novel M2 subset, termed M2d [61]. M2d macrophages exhibit a typical M2 cytokine production profile (IL-10^high^IL-12^low^) [9,61]. Furthermore, they present a CD14^high^ CD163^high^ TGF-β^high^ CD86^low^ ILT2^high^ ILT3^high^ phenotype and produce high levels of CCL18, but low levels of CCL1, CCL17, CCL22, TNFα, and PTX3. Moreover, they display poor T cell co-stimulatory activity and suppress T cell proliferation [9,61].

**Figure 4:**
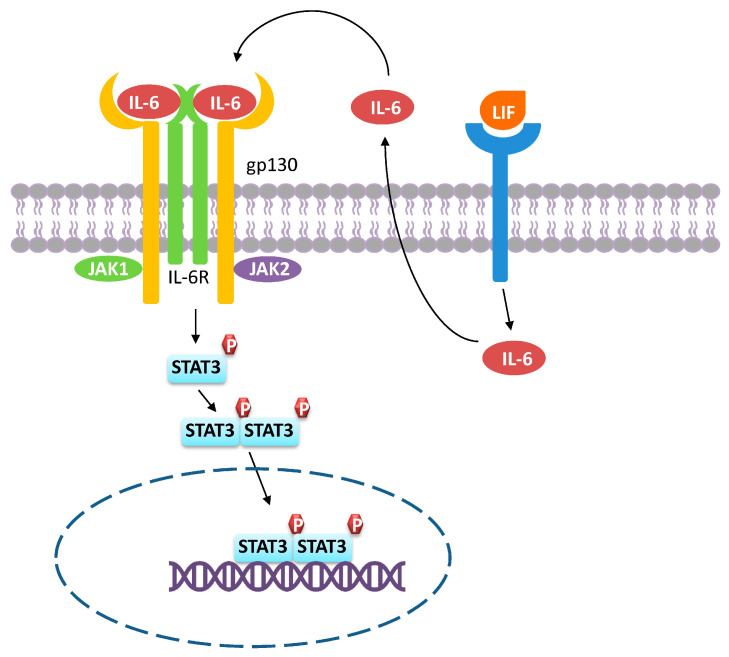
M2d polarization. The binding of IL-6 to the IL-6R/gp130 receptor complex leads to the recruitment of JAK1/2, which in turn phosphorylates STAT3, leading to STAT3 dimerization and translocation into the nucleus, where it activates gene transcription. LIF belongs to the IL-6 family and binding of LIF to its receptor results in IL-6 production. Thus, M2d macrophages can consume IL-6 in an autocrine manner.

IL-6 is a pleiotropic cytokine secreted by many cells including lymphocytes, fibroblasts, monocytes/macrophages, and tumor cells [84]. IL-6 is implicated in various tumor types for promoting tumor growth such as ovarian cancer [61], breast cancer [85], gastric cancer [9], colorectal cancer [86] as well as hepatocellular cancer [84]. IL-6 induces M2d differentiation by activating STAT3 mediated cell signaling [9], resulting in the induction of a series of downstream molecules (Figure 4). To initiate cell signaling, IL-6 first binds to the IL-6 receptor (IL-6R), which is usually found in many cells including monocytes/macrophages and neutrophils. The engagement of IL-6 to its receptor, IL-6R, induces conformational changes in the intracellular domains and dimerization of IL-6R. This further triggers the recruitment of gp130 to assemble a functional tetramer in which each IL-6/IL-6R complex is associated with one gp130 molecule. Subsequently, the IL-6/IL-6R/gp130 complex recruits and activates JAKs. Recruited JAKs then activates STAT3 via phosphorylation. Phosphorylated STAT3 proteins dimerize and translocate into the nucleus, inducing the transcription of pro-inflammatory genes [84,87]. During M2d polarization, macrophages can consume M-CSF in an autocrine manner where IL-6 and LIF play a role in promoting such M-CSF consumption. Both IL-6 and LIF enhance M-CSF uptake and induce macrophages to fully differentiate into the M2d phenotype. In addition, LIF increases the production of IL-6 in macrophages, which further enhances the ability for macrophages taking up M-CSF [61].

#### Pro-Tumoral Effects of M2d Macrophages

**Figure 5:**
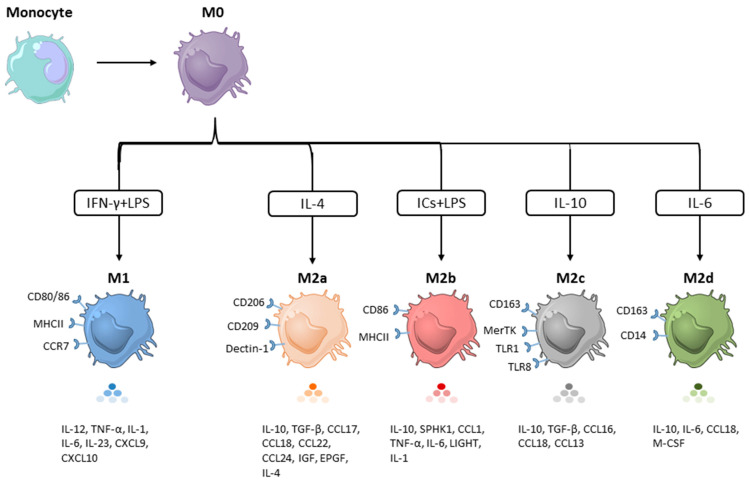
A graphic overview of macrophage polarization and their characteristics. Macrophages are first differentiated from their precursor monocytes to uncommitted M0 macrophages. In response to different stimuli, M0 macrophages can further polarize into distinct subsets with characteristic phenotypic markers and cytokine production profiles.

In relation to cancer, M2d macrophages promote tumor progression through two main mechanisms. First, M2d cells can produce pro-tumoral factors. For example, M2d macrophages were able to promote cancer cell proliferation and migration by secreting IL-10 and TGF-β in gastric cancer [9]. Additionally, VEGF and MMP9, produced by M2d, are expected to induce angiogenesis and degradation of the extracellular matrix, facilitating tumor metastasis [61]. As described above, M2d cells are a source of IL-6 that is implicated in various tumor types such as ovarian cancer [61], breast cancer [85], gastric cancer [9], colorectal cancer [86] as well as hepatocellular cancer [84]. The IL-6/JAK/STAT3 canonical pathway regulates the expression of several genes linked to anti-apoptosis, angiogenesis, metastasis, proliferation, and drug resistance. It is therefore not surprising that the IL-6-STAT3 pathway is now considered as a major target for the treatment of cancer [84]. M2d can also dampen normal immune responses, supporting the tumor cell evasion from immune surveillance. M2d is known to express several molecules related to immune suppression such as the IDO, IL10, Siglec 15, and PD-1 ligands [61]. All M2 subsets are poor in stimulating T cells. In conclusion, the different M2 macrophage subsets have in common the effects of tumor promotion and the suppression of effective adaptive immune responses.An overview of macrophage subsets and their phenotypic markers is shown in Figure 5. Table 1 summarizes the role of macrophage subsets in various cancer types.

## 3. TAMs as a Target for Cancer Therapy

It appears that there is a consensus in the literature that M2 macrophages in the TME support tumor growth and therapy resistance in most cancer types. These findings provide a strong foundation for targeting these cells and/or their progenitors to improve patient outcomes. Currently, the therapeutic strategies fall into four categories: (1) inhibiting the recruitment of monocytes/macrophages, (2) restoring macrophage phagocytic activity, (3) reprogramming M2 into anti-tumoral M1 phenotype, and (4) depleting monocytes/TAMs.

### 3.1. Blocking Monocyte/Macrophage Recruitment to Tumors

Monocytes/macrophages are recruited to the tumor site by the factors secreted from tumor cells and tumor stromal cells. Infiltrating monocytes/macrophages are then educated by the TME and differentiate into TAMs, serving as a continuous supplement of TAMs. Signaling pathways that are involved in monocyte recruitment are thus the main targets in interfering monocyte/macrophage recruitment. Various studies have demonstrated that the CSF-1/CSF-1 axis is the primary regulator of monocyte/macrophage recruitment and differentiation. CSFR1 is expressed on the cell surface of monocytes and macrophages, whereas CSF1 (also known as M-CSF) is secreted by tumor cells to recruit and polarize monocytes into TAMs. Currently, several small molecules and antibodies targeting the M-CSF/CSF1R axis have been developed. They are usually used alone or in combination with other therapeutic modalities (e.g., checkpoint immunotherapy, radiotherapy, or chemotherapy) for treating certain types of solid tumors. PLX3397 (also named pexidartinib) is a small molecule that received FDA approval in 2019 for treating tenosynovial giant cell tumor, where M-CSF/CSF1R is the major factor triggering oncogenic transformation [11,13]. By targeting CSF1R, PLX3397 inhibited the recruitment of macrophages, but also restored T cell infiltration into the TME [11,13,15]. Of note, PLX3397 is also a two-edged sword because it can trigger side effects such as anemia, neutropenia, and hypertension [94]. This is due to the expression of CSF1R on cells other than monocytes/ macrophages such as neutrophils and Kupffer cells (liver-resident macrophages). The monoclonal antibody RG7155 is another example of a CSF1R inhibitor; it binds to CSF1R and interferes with its dimerization, impeding signal transduction. RG7155 was able to deplete both monocytes and macrophages and alter the composition of T cells in the TME in patients with diffuse-type giant cell tumor. The use of an RNA interference strategy to inhibit the expression of CSF1R or CSF1 also affected tumor growth in both xenograft and GEM models of cancers [95,96,97]. Overall, CSF1R inhibitors are useful in treating tumors with a high expression of M-CSF, but they can also cause systemic symptoms due to the non-selective depletion of monocytes/macrophages.

The CCL2/CCR2 axis is another target that is critical in monocyte mobilization. In the context of tumors, CCL2 produced by tumor cells and/or tumor stromal cells is released into the circulation and engages with CCR2 expressed on monocytes. CCL2 tagged monocytes are thus mobilized and recruited to the TME, resulting in the polarization to TAMs. To date, there are two therapeutic modalities for CCL2/CCR2 axis blockage: one is the anti-CCL2 antibody (i.e., carlumab), and the other is the CCR2 antagonist (i.e., PF-04136309). Both carlumab and PF-04136309 are under clinical trials in combination with other therapeutic modalities. In comparison to carlumab, PF-04136309 has provided more promising results as it showed a reduction in the circulating CCR2+ monocytes and TAMs accompanied with an increased number of bone marrow CCR2+ monocytes [11,15].

### 3.2. Restoring Macrophage Intrinsic Functions

Macrophages are immune cells with multi-functions including phagocytosis, antigen presentation, T cell activation, induction of apoptosis, etc. However, the TME hijacks and educates macrophages and polarize them to TAMs, which lost most of their normal intrinsic functions. Considering TAMs are highly plastic cells, this property can thereby be exploited to restore their anti-tumor functions. Currently, several strategies are being investigated.

#### 3.2.1. Enhancement of Phagocytosis by Blocking the CD47/SIRPα Axis

Bone marrow derived macrophages can infiltrate the TME and engulf tumor cells efficiently. Nevertheless, this ability is soon lost once they differentiate into TAMs. The restoration of such phagocytic ability can be achieved by blocking the CD47/signal regulatory protein-α (SIRPα) axis. CD47 is a transmembrane protein that is widely expressed in all types of cells, and is especially overexpressed in most tumor cells. SIRPα is the receptor for CD47, and is mainly expressed on myeloid cells including dendritic cells and macrophages. The binding of CD47 to SIRPα expressed on macrophages prevents the phagocytosis of tumor cells. Thus, CD47 is regarded as a ‘do not eat me’ signal sent by tumor cells to macrophages to avoid being phagocytosed. Therefore, blocking the CD47/SIRPα axis can enhance the phagocytic properties of macrophages. Such blockades have been found to have several benefits for cancer treatment such as M2 repolarization, the enhancement of DC-antigen presentation, induction of apoptosis in tumor cells, and the activation of NK cell-mediated antigen-dependent cellular cytotoxicity (ADCC). Currently, several antibodies as well as CD47 targeting peptides are being developed to interfere with the CD47/SIRPα axis [11,13,15,98,99,100] (Table 2).

The use of anti-CD47 antibodies is the most common strategy for blocking the CD47/SIRPα axis. The IgG Fc regions of anti-CD47 antibodies can bind to Fcγ receptors displayed on macrophages to induce antibody-dependent cellular phagocytosis (ADCP), thereby synergistically enhancing phagocytosis triggered by CD47/SIRPα blockage. In combination with immunotherapies (i.e., anti-PD1, anti-EGFR), the blockage efficacy can be enhanced. As an example, Hu5F9-G4 (also named Magrolimab) is a humanized anti-CD47 antibody for the treatment of advanced solid tumors, lymphoma, pediatric brain tumor, and leukemia. Hu5F9-G4 contains two domains: one is a SIRPα analog that can interfere with the interaction between CD47 and SIRPα, and the other is an Fc region that enhances phagocytosis via ADCP. The clinical efficacy and safety of Hu5F9-G4 used alone for treating solid tumors and hematological malignancies have been evaluated in Phase I clinical trials (ClinicalTrials.gov Identifier: NCT02216409 and NCT02678338), and in combination with Cetuximab (an anti-EGFR antibody) in a Phase I/II clinical trial (ClinicalTrials.gov Identifier: NCT02953782) for treating solid tumors and advanced colorectal cancer. The concerns regarding bringing Hu5F9-G4 into the clinics are the observed side effects such as headache, nausea, and anemia. Hence, the safety of CD47/SIRPα axis inhibitors needs careful investigation. However, aside from the benefits, the application of anti-CD47 antibodies is accompanied with some side effects. This is because in contrast to SIRPα, which is restricted to the cell surface of myeloid cells, CD47 is widely expressed in all types of cells, especially neurons and red blood cells (RBCs) [11,13,15,98,99]. Dedieu’s group engineered a peptide, named TAX2, that targets the interaction between tumor-overexpressed thrombospondin-1 (TSP-1) and CD47. TSP-1 is involved in inflammation, immune responses, and the modulation of tumor growth [101,102]. Interestingly, the TAX2 peptide inhibited tumor angiogenesis in several experimental cancer models [103,104].

**Table 2:** Therapeutic strategies targeting TAMs.

Strategy	Examples		Benefits	Side Effects	Ref.
**Inhibition of monocyte/** **macrophage recruitment**	M-CSF/CSF1R blockade	Small molecules: PLX3397, PLX7486, JNJ-40346527, ARRY-382, BLZ945, CS2164	Eliminate circulating monocytes/TAMs and restore T cell infiltration	Systemic symptoms including Anemia; neutropenia; hepatotoxicity; hypertension	[11,13,15]
		Monoclonal antibodies: RG7155, IMC-CS4, R05509554, RG7155, FPA008, AMG820, LY3022855, PD-0360324			
	CCL2/CCR2 blockade	CCL2 inhibitor (Carlumab) CCR2 inhibitor (PF-04136309)	Eliminate circulating monocytes/TAMs	No effect on resident TAMs; relapse; pulmonary toxicity	[23]
**Depletion of monocytes/** **macrophages**	Inorganic compounds: Bisphosphonates and its derivatives		Useful in killing monocytes, macrophages, and tumor cells in bone metastases	Short plasma half-life, rapid kidney clearance	[11,15,105]
	Chemical agents: Trabectedin, lurbinectedin		Cytotoxic to tumor cells, monocytes, TAMs, and MDSCs	Anaphylaxis, neutropenic sepsis, rhabdomyolysis, hepatotoxicity, cardiomyopathy, capillary leak syndrome, and tissue necrosis	[11,13,15,106]
	M2 targeting peptides: NW, M2pep, UNO, Melittin, RP182, LyP-1		Highly specific; drug delivery and imaging; less immunogenic; cheap and easy to synthesize; high penetration rate	Unstable bio-stability	[11,89,90,91,92,107,108,109,110,111,112,113,114,115,116,117,118,119,120,121,122,123,124,125,126,127,128,129,130,131]
**Restoring** **macrophage intrinsic** **functions**	Enhance phagocytosis	CD47 targeting peptides: Anti-CD47 antibodies: Hu5F9-G4, CC-90002, SRF231, IBI188, ZL1201, TTI-621, ALX148	Enhance macrophage functions (phagocytosis, antigen presentation, ADCC); induce tumor cell apoptosis; repolarize TAMs towards M1	Anemia; nausea; headache; isolated neutropenia	[11,13,15]
		pep-20, pep20-D12, TAX2			[100,101,102]
		Anti-SIRPα antibodies: Velcro-CD47, KWAR23, ADU-1805			[98]
	Enhance antigen presentation	Anti-CD40 antibodies: CP-870,893, ChiLob7/4, RO7009789	Prime T cell activation; promote anti-tumoral factors secretion; alter M2 cytokine profile	Cytokine release syndrome; liver toxicity	[11,13,15,105,132,133]

Pep-20 is a short peptide that targets both human and murine CD47 molecules. Pep-20 was able to block the interaction between CD47 and SIRPα, thereby enhancing the macrophage-mediated phagocytosis of tumor cells. Moreover, pep-20 treatment reduced the tumor size, promoted the infiltration of cytotoxic T cells, and prolonged the overall survival in mice bearing colon adenocarcinoma. Furthermore, no reduction in RBC number was observed, suggesting that pep-20 is a safer therapeutic candidate, considering the blood toxicity commonly observed with CD47/SIRPα blockade [100].

#### 3.2.2. Enhancement of Antigen Presentation via Enhancing CD40/CD40L Interaction

CD40 is expressed on antigen-presenting cells such as macrophages, monocytes, DCs, and B cells, whereas its ligand (CD40L) is expressed by T cells, B cells, NK cells, basophils, and mast cells. The interaction between CD40 and CD40L is required for effective T cell priming by upregulating the expression of MHC molecules and the secretion of pro-inflammatory factors. Notably, enhanced CD40/CD40L interaction can induce a switch from the cytokine production profile in TAMs to an M1-like cytokine production profile and promoted the secretion of various factors (i.e., NO and TNF-α) [105,133]. This effect suppressed tumor growth by inducing T cell activation as well as triggering the release of various factors with tumor suppressing or killing functions. The CD40/CD40L engagement is being exploited in cancer therapy and several anti-CD40 antibodies have been developed and tested in clinical trials (Table 2). Anti-CD40 antibodies are usually combined with other therapeutic modalities including immunotherapy, chemotherapy, and targeted therapy in treating solid tumors [11,13,15,132]. Of note, aside from the efficacy, unwanted side effects have been observed, with cytokine release syndrome being of particular concern. To circumvent this, the local administration of anti-CD40 antibodies to enhance CD40/CD40L interaction was suggested [105].

#### 3.2.3. CAR-Macrophages

An increasing number of chimeric antigen receptor (CAR) therapies are being developed and tested in clinical studies including CAR-T cells and CAR-NK cells. CAR-T cells, which are engineered to recognize tumor-specific antigens, are successful in some blood cancers, but have not been effective in solid tumors [134,135,136]. Therefore, researchers have been investigating the use of CAR technology on macrophages. Macrophages have the ability to penetrate solid tumors, ingest malignant cells, and stimulate adaptive immunity, making macrophage therapy quite different to CAR-T cells or CAR-NK cells [134,135]. While T cells and NK cells typically kill tumor cells through granzyme-induced damage, macrophages tend to kill by engulfing and digesting tumor cells. CAR is a recombinant chimeric antigen receptor that typically targets native cell surface antigens and, unlike T cell receptors, the recognition by CAR is not MHC-dependent [135]. Klichinsky et al. reported that human macrophages transduced with CARs exhibited a sustained M1 phenotype and demonstrated potent antigen-specific phagocytosis of tumor cells in vitro [137]. They also showed that in humanized mouse models, CAR macrophages activated immature dendritic cells and enhanced the recruitment of activated CD8^+^ T cells to tumor site, thus enhancing the overall antitumor response [137]. Furthermore, these CAR-macrophages significantly reduced the tumor burden and prolonged the overall survival in immunodeficient ovarian cancer xenograft mouse models [137]. In contrast to CAR-T cells, CAR-macrophages can induce a pro-inflammatory milieu by recruiting and activating other immune cells such as DCs, T cells, and NK cells [134].

Despite the merits offered by CAR-macrophages, one major challenge for equipping macrophages with CARs is transgene delivery. Macrophages express a large number of nucleic acid sensors, thereby making transfection challenging. Often, nucleic acids activate the interferon pathway in macrophages, leading to the degradation of exogenous DNA/RNA and/or killing of the macrophages [138]. To circumvent this, Klichinsky et al. identified a replication-incompetent chimeric adenoviral vector Ad5f35 as a good vehicle in delivering CAR gene constructs into primary human macrophages [137]. In addition to successful genetic manipulation, the use of the Ad5f35 virus not only skewed the host primary human macrophages toward the M1 phenotype, but also induced a pro-inflammatory state in the TME of a humanized mouse model [137]. In a second study, Chen et al. used a superstructure named a nanoporter-hydrogel that was able to deliver the CAR gene to macrophages and microglia locally upon intratumoral injection [139]. The engineered CAR targeted CD133 is a protein that is highly expressed in glioblastoma and is also considered as a marker for glioma stem cells. Interesting, the engineered CAR was able to block tumor recurrence in a mouse model for glioblastoma. The treatment was associated with an increase in the number of CD8^+^ T cells and M1 macrophages, and a decrease in the number of M2 macrophages in tumors [139]. This non-viral CAR delivery strategy facilitates the local delivery of CARs to macrophages, thereby avoiding the side effects introduced by conventional systematic infusion.

### 3.3. Reprogramming of TAMs

**Figure 6:**
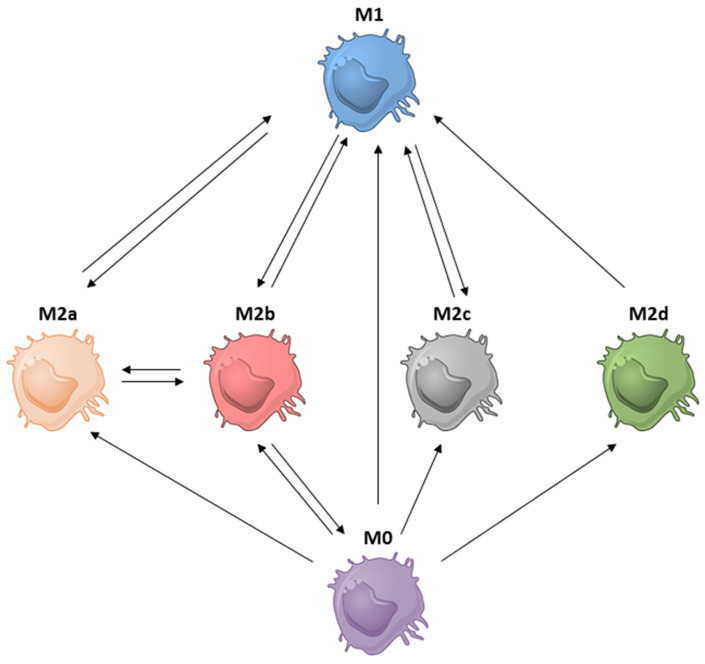
Relationship between the different macrophage subsets. For details see the text.

Phenotypically and functionally distinct macrophage subsets co-exist in the TME. Macrophages change their phenotypic and functional activities according to the stimuli they receive from the TME. This property can thus be exploited to reprogram tumor supportive M2 macrophages into anti-tumoral M1 macrophages. As indicated above, each M2 subset is polarized by certain stimuli that trigger particular cell signaling pathways. Therefore, M2 may be reverted to M1 macrophages by interfering with the activities of these stimuli or by inhibiting particular cell signaling pathways. The known conversions between macrophage subsets are summarized in Figure 6 and Table 3. As depicted, macrophages can convert between different M2 subsets or revert to M1 macrophages under certain conditions. For instance, interfering STAT6-mediated signaling can be used to reprogram M2a back to M1 (Figure 6).

As an example, PD-1 deficiency was shown to suppress M2a polarization by reducing STAT6 signaling [3] while promoting M1 polarization by enhancing the STAT1 signaling pathway [93]. Moreover, some TLR agonists such as CpG and R848 have the potential to re-educate M2 into M1 macrophages. In this respect, Patrícia et al. showed that M2 macrophages could be reverted into M1 in vivo when nanoparticles loaded with R848, a TLR7/8 agonist, were delivered to M2 macrophages via a M2-targeting peptide [92]. The effect of R848 is more likely due to its capacity to activate MyD88, a central TIR domain adaptor molecule responsible for all TLR-mediated signaling. Similarly, Anderson et al. utilized corosolic acid to inhibit STAT3-mediated signaling in IL-10-stimulated human monocyte-derived M2c and successfully reverted M2c to M1 macrophages. The repolarized macrophages exhibited an M1-like cytokine production profile (IL-12^high^, IL-10^low^, IFN-γ^high^, and TNF-α^high^) [79].

### 3.4. Depletion of Monocytes/TAMs

Macrophages were found to be the major myeloid cell types infiltrating tumor tissues and in most cases, they accounted for approximately half of the tumor mass [13,22]. Their presence in the TME correlated with poor prognosis in the breast, ovarian, lung, oral, bladder, gastric, Hodgkin lymphoma, and thyroid cancers [15]. Several preclinical and clinical studies have shown that reducing the number of TAMs can repress tumor progression and improve treatment outcomes [10,114,115,144,145,146,147]. Furthermore, as TAMs are mainly replaced by circulating monocytes, depleting monocytes is also a way to reduce the number of TAMs. The current available strategies applied for depleting monocytes/TAMs fall into three categories: bisphosphonate, trabectedin, and peptides.

#### 3.4.1. Biophosphonate

Bisphosphonate is a type of inorganic compound that has been found to kill both monocytes/macrophages and tumor cells via inducing apoptosis. It can also inhibit angiogenesis and tumor cell invasion [11,13,105]. Since it shares an identical structure with pyrophosphatases in the bone matrix, bisphosphonate can rapidly be taken up by osteoclasts. This property makes it especially effective in treating cancers with bone metastasis [11,15,105]. Moreover, the killing efficacy of bisphosphonate relies on the phagocytic activities of macrophages. However, the short plasma half-life, rapid kidney clearance, and causative severe side effects limit the clinical use of bisphosphonates. To overcome these shortcomings, liposomes and nanoparticles have been used to encapsulate bisphosphonates. In addition to reducing the side effects, the encapsulation extends the plasma retention time as well as passively accumulates the packaged bisphosphonates in the TME through the enhanced permeability and retention (EPR) effect [11,15,105]. The encapsulated bisphosphonates are more easily taken up by macrophages, leading to macrophage eradication [15,105]. For example, clodroplip is formed by clodronate encapsulated by liposomes, which is one of the most commonly-used bisphosphonates. It has been reported that clodroplip reduced tumor mass and interfered with TAM infiltration in lung cancer with bone metastasis [15]. Overall, bisphosphonates are potent compounds for treating bone metastases, but they need to be encapsulated to optimize the therapeutic potency and reduce the side effects.

#### 3.4.2. Trabectedin

Trabectedin was originally extracted from the sea squirt *Ecteinascidia turbinata* and preclinical studies demonstrated activity at low concentrations against a variety of tumors [15]. It was approved in 2007 as Yondelis^®^ (Pharma Mar S.A. and Johnson and Johnson). The drug is now used for treating advanced soft tissue sarcoma and relapsed ovarian cancer [11,15]. Trabectedin not only kills tumor cells via a process called alkylation, but it can also induce caspase 8-mediated apoptosis in monocytes/macrophages by activating TNF-related apoptosis-inducing ligand receptors (TRAILR) [11,13,15]. Additionally, trabectedin has no effect on neutrophils, lymphocytes, and normal resident macrophages as they only express decoy TRAILR on the cell surface, whereas monocytes and macrophages express functional TRAILR [13,15]. Thus, monocytes and macrophages can be selectively eliminated by trabectedin without disturbing normal cells [13]. MDSCs are also susceptible to trabectedin [13]. However, aside from these benefits, anaphylaxis, neutropenic sepsis, rhabdomyolysis, hepatotoxicity, cardiomyopathy, capillary leak syndrome, and tissue necrosis have also been reported [106].

#### 3.4.3. Targeting Peptides

Over the years, several peptides have been developed to selectively target TAMs, many of which were selected via the phage display technique (Table 4) [11,148]. In comparison to antibodies, they are easier to synthesize, are less expensive, have high tumor penetration potential, and are less immunogenic. In addition, peptides can function as carriers to deliver therapeutic agents to target cells and can be used as imaging agents when conjugated with fluorescent dyes. Depending on the applications, their binding affinity and specificity, serum stability, and blood availability can be optimized by amino acid or chemical modifications [11].

The affinity selection of peptide libraries on monocyte-derived dendritic cells resulted in the identification of a peptide named NW that showed specific binding to monocytes, dendritic cells, and macrophages [88]. Recently, the peptide was shown to bind prohibitin 1 (PHB1) displayed on the cell surface of human monocytes/macrophages with high affinity [108,109,110]. Although PHB1 is also expressed on other cell types such as T cells, the results suggest that NW peptide binding to PHB1 is monocyte/macrophage specific. The conjugation of lytic peptides to the NW peptide led to the design of fusion peptides that killed macrophages [108,109,110]. However, along with the merits, NW peptides also target cells other than TAMs (e.g., DCs and M1) [108,109,110]. Therefore, further modifications to optimize its binding specificity and safety are needed.

M2pep is another peptide selected from random peptide libraries [111]. M2pep selectively binds to M2a polarized from primary murine bone marrow-derived macrophages with higher affinity than that to M1 [111]. Furthermore, M2pep has been used as a carrier to deliver various cargos into the cells including lytic peptide (i.e., KLA peptide), CSF1R inhibitor PLX3397, and siRNAs [111,147]. The treatment of mice with nanoparticles loaded with PLX3397 and coated with M2pep inhibited tumor growth [147]. The expression of the M2pep receptor, yet to be identified, varied in different tumors, limiting the universal use of such peptides [149]. To increase its therapeutic potency, the peptide was cyclized and modified by substituting lysine at position 9 and tryptophan at position 10 with arginine and tyrosine, respectively. Such changes led to an increase in serum stability and enhanced binding affinity [112,113]. Similar to the NW peptide, M2pep also targets normal tissue resident macrophages [149].

The UNO peptide was selected from a cyclic peptide phage library in vivo. However, this cyclic peptide must be converted into a linear peptide in order to facilitate its binding to target cells. The receptor for the linear UNO peptide was identified as CD206 and the binding was specific, as no binding to other cell types including M1 macrophages was observed. Linear UNO peptide efficiently infiltrated the TME where it selectively and exclusively bound to CD206+ M2 macrophages, regardless of tumor types (i.e., glioblastoma, gastric carcinoma, melanoma, breast cancer, and its lung metastasis). Like the NW peptide and M2pep, the linear UNO peptide and its derivatives can navigate various cargos to TAMs (i.e., paxlitaxel or TLR7/8 agonist loaded nanoparticles) and be used for imaging (i.e., when coupled to 5(6)-carboxyfluorescein (FAM)) [90,91,92,107].

Melittin is a natural polypeptide composed of 26 amino acid residues and the major component of bee venom. It can kill tumor cells by inducing apoptosis or disrupting cell membranes. Melittin preferentially bound to human/murine CD206+ M2 macrophages rather than M1 macrophages and other leukocytes [90]. Within the safe dose range, melittin alone or fused with lytic peptide (i.e., KLA peptide) inhibited tumor growth, suppressed angiogenesis, and reduced the number of M2 macrophages in mice bearing lung carcinoma or melanoma [114,115].

LyP-1 peptide was selected from a cyclic peptide phage library ex vivo and in vivo. It can home to tumor lymphatics and target tumor cells as well as pathological macrophages (i.e., TAMs, atherosclerotic plaque macrophages) [118,119,122]. Intriguingly, LyP-1 peptide has two receptors. The first receptor, p32, is expressed on the cell surface and conducts the peptide binding [120]. The bound LyP-1 peptide is then subjected to proteolytic cleavage, during which the cyclic peptide is converted into a linear peptide and exposes the (R/K)XX(R/K) motif, which is required by the peptide to be recognized by the second receptor, neuropilin-1/2 (NRP1/2), leading to peptide internalization [123]. Such an internalization property endows the LyP-1 peptide with the ability to serve as a delivery vehicle or function as an imaging agent. In addition, the LyP-1 peptide has an intrinsic pro-apoptotic ability that makes it more unique [118,119,121,125]. Although the LyP-1 peptide has become a widely studied peptide due to its satisfactory homing, internalization, and pro-apoptotic properties, the presence of disulfide bonds is expected to reduce its in vivo stability. In this respect, Li et al. replaced the cysteine with selenocysteine, leading to enhanced serum stability and function [124].

Apart from the M2 targeting peptides described above, others are listed in Table 4. Compared to bisphosphonate and trabectedin, which non-selectively target M2 macrophages, peptides are more suitable for targeting macrophages. With proper modifications, peptides with high specificity and affinity can be developed to selectively target M2 macrophages, thus avoiding the systematic depletion of normal macrophages and other cells. Moreover, peptides can serve as carriers to transport therapeutics to TAMs and exert killing activity in a spatiotemporal manner. By conjugating drugs that can reprogram TAMs to targeting peptides, M2 macrophages may be reverted to M1 macrophages with anti-tumoral function.

Compared to antibodies, peptides are cheaper and easier to synthesize, and their small size allows for a better tissue penetration. Since the small size of peptides allows for diffusion into tumor tissues, peptides that show internalization into TAMs could function as effective drug-delivery agents. However, a drawback to peptides in therapeutics has been their short half-life in circulation. Their small size leads to a rapid excretion through the kidneys, and in addition, peptides are also vulnerable to enzymatic cleavage. However, several approaches for extending the serum half-life have helped in overcoming these drawbacks to optimize peptides for both therapeutic and diagnostic use. For example, degradation by proteases can be prevented through chemical modifications in the amino acid sequences [150]. Similarly, fusing biologically active peptides with the Fc domain of IgG increased their half-life and therapeutic efficacy [151]. The Fc domain is important for the recruitment of immune effector cells such as NK cells to kill target cells via the induction of ADCC. Although not every identified M2 targeting peptide exclusively targets TAMs, they still hold great therapeutic potential.

## 4. Conclusions and Future Directions

Cancer immunotherapy has already gained a prominent role in the treatment of cancer, with checkpoint inhibitors being the most promising therapeutic antibodies developed thus far. However, the development of an immunosuppressive TME constitutes one of the major cancer-evasion strategies. TAMs, the most abundant inflammatory cell population in the TME, play an essential role in tumor immune regulation. TAM activation is typically characterized by two polarized states, M1 and M2, which differ in the ways in how they affect tumors. Both phenotypes are dynamic and depend on the cytokines and growth factors present in the TME. Additionally, there is a substantial difference in the receptor and signaling pathways of the stimuli leading to M1 and various M2 subtypes. In contrast to M1 macrophages, M2 macrophages support tumor growth, immune evasion, and metastasis in most solid tumors. Without a doubt, M2 macrophages have become an interesting therapeutic target in cancers based on these observations. Essentially, one would like to keep or increase the number of M1 macrophages, while suppress or reduce the number of M2 macrophages. This could, in principle, be achieved by either killing M2 macrophages or suppressing the recruitment of circulating monocytes and MDSCs to tumor tissues. Targeting only intra-tumoral TAMs may not be sufficient to achieve therapeutic benefits. Indeed, constant replenishment from circulating monocytes and M-MDSCs may maintain the TAM pool in tumors. Although it has been shown that TAM depletion improves the T cell immune response and delays tumor progression, it is not clear whether it is caused by the systemic depletion of monocytes/other immune suppressor cells or solely the depletion of TAMs. Is it sufficient to delay tumor growth simply by blocking monocyte recruitment or by decreasing the TAM pool? The answer to this question is important for the design of more effective treatments. There are now various methods to target circulating monocytes and prevent them from migrating to tumors. One of these methods is the use of receptor-targeting peptides. Peptide-based nanostructures have a wide range of biomedical applications including drug delivery, biological imaging, and targeted therapies. With respect to immune suppressor cells, the molecular mechanisms regulating the conversion of M-MDSC to TAMs are not yet known. Whether the migration route of MDSCs to the tumor site is similar to that of monocytes is also unclear. Additionally, effective and safe treatment require well-timed interventions as well as a better understanding of the molecular mechanisms by which TAMs can rapidly switch their phenotypic and functional properties. We should also take into consideration the metabolic fluctuations in macrophages that are linked to their phenotype and function [152]. For example, tumor cell-derived lactate drives macrophage differentiation toward M2-like macrophages. Hence, some metabolic interventions may be harnessed in combination with other therapeutic agents. Targeting tumor-infiltrating macrophages, a cell type involved in various chronic diseases including cancer, heart failure, and atherosclerosis [153,154,155], should have broad therapeutic applications.

## Figures and Tables

**Table 1:** Macrophage subsets and roles in cancers.

Macrophage Subset	Stimulators	Phenotypic Markers	Secreted Molecules	Produced Enzymes	Other Molecules	Functions in Cancer	Associated Cancer Types
M1 (classical activated macrophages)	LPS and IFN-Ƴ	CD80/86^high^, MHCII^high^, TLR2, TLR4, CCR7^high^	IL-12, TNF-α, IL-1, IL-6, IL-23, CXCL9, CXCL10	iNOS	GAS5	Inhibit cancer growth and progression (in general)	Ovarian cancer (M1 promoted cancer cell invasion ability via NFκB-mediated signaling [88])
M2a (wound-healing macrophages)	IL-4 and/or IL-13	CD206^high^, CD209^high^, Dectin-1^high^, CD163^low–medium^, CD86^low^, CD14^low–medium^, IL-1R	IL-10, TGF-β, CCL17, CCL18, CCL22, CCL24, IGF, EPGF, IL-4	Arg1	GAS5, GAS6	Tissue repair, metastasis [7], invasion [7,89], promote tumor cell proliferation [3,89]	Lung cancer [2,3], breast cancer [4,7,90,91,92], melanoma [89,91], glioma [91], gastric carcinoma [91], cervical cancer [18]
M2b (regulatory macrophages)	IC and TLR agonists (i.e., LPS)/IC and IL-1R agonists (i.e., IL-1β)	CD163^low^, CD86^medium–high^, MHCII^high^, CD14^medium^	IL-10, SPHK1, CCL1, TNF-α, IL-6, LIGHT, IL-1	iNOS, SPHK1	No GAS5 expression	Promote tumor growth [8], metastasis [5]	Hepatocellular carcinoma [8,93], bevacizumab resistant triple-negative breast cancer [5], breast cancer [4]
M2c (acquired deactivation macrophages)	IL-10, glucocorticoids, TGF-β	CD163^high^, CD14^medium^, CD206^low–medium^, CD86^medium^, MerTK^medium–high^, CD16, TLR1, TLR8	IL-10, TGF-β, CCL16, CCL18, CCL13	Arg1	GAS6	Phagocyte apoptotic cells [20]	Lung cancer [2], breast cancer [4], mouse melanoma, and lymphoma [10]
M2d (tumor-associated macrophages)	IL-6/TLR ligands and A2 adenosine receptor agonists (i.e., LIF)	CD163^high^, CD86^low^, CD14^high^	IL-10, IL-6, CCL18, M-CSF	iNOS		Angiogenesis and metastasis	Ovarian cancer [61], gastric cancer [9]

**Table 3:** Conversions between the macrophage subsets based on the published data.

Category	Strategies	Ref.
M1 to M2a	Promote STAT6 signaling: PD-1 exposure	[93]
M1 to M2b	Crosslinking Fcγ receptors by ICs	[21,53]
M1 to M2c	Deficiency in Pellino-1 with IL-10 induction	[10]
M2a to M1	Inhibit STAT6 signaling: (1) PD-1 deficiency; (2) STAT6 deficiency); TLR agonists: (1) TLR7/8 agonist: R848 (resiquimod) (2) TLR3 agonist: poly(I:C); (3) TLR7 agonist: imiquimod	[3,92,93,140,141]
M2a to M2b	Exposure to LPS and ICs	[21]
M2b to M0/M1	CCL1 depletion or inhibition	[8,54,56,62]
M2b to M2a	PPARƳ ligands	[142]
M2d to M1	IFN-γ exposure	[66,143]
M2c to M1	STAT3 inhibition: corosolic acid	[79]

**Table 4:** Examples of TAM targeting peptides.

Name	Peptide Sequence	Receptor	Targets	Methods	Ref.
NW	NWYLPWLGTNDW	PHB1	Human DCs, monocytes, M1, M2	In vitro phage display	[108,109,110]
M2pep	YEQDPWGVKWWY	Unknown	Murine bone marrow monocytes derived M1 and M2	In vitro phage display	[111,112,113,147,149]
UNO	CSPGAKVRC	CD206	Human and murine CD206+ M2 macrophages	In vivo phage display	[90,91,92,107]
Melittin	GIGAVLKVLTTGLPAL-ISWIKRKRQQ	Unknown	Human and murine CD206+ M2 macrophages, tumor cells, endothelial cell, red blood cells	Identified from honey bee venom	[89,114,115]
LyP-1	CGNKRTRGC	p32, NRP 1/2	Tumor lymphatics, tumor cells (i.e., MDA-MB-435), TAMs, atherosclerotic plaque macrophages	In vitro and in vivo phage display	[118,119,120,121,122,123,124,125]
RP182	KFRKAFKRFF	CD206	Human and murine CD206+ M2 macrophages	In silico biophysical homology screening	[116,117]
T4	NLLMAAS	Tie2 receptor	Tie2 expressing human endothelial cells and TAMs	In vitro phage display	[126,127]
CRV	CRVLRSGSC	Retinoid X receptor beta	Murine and human macrophages	In vitro phage display	[130,131]
IL4RPep-1	CRKRLDRNC	IL-4 receptor	IL-4 receptor expressing murine T4 breast cancer cells and TAMs	Ex vivo phage display	[128,129]
Pep-20	AWSATWSNYWRH	CD47	Myeloid cells (i.e., DCs, monocytes, macrophages)	In vitro phage display	[100]

## Data Availability

Not applicable.

## Abbreviations

ADCC	Antigen-dependent cellular cytotoxicity
ADCP	Antibody-dependent cellular phagocytosis
ALD-DNA	Activated lymphocyte derived DNA
APC	Antigen presenting cell
Arg1	Arginase 1
BMDM	Bone marrow-derived macrophages
CAR	Chimeric antigen receptor
DC	Dendritic cell
ECM	Extracellular matrix
EPR	Enhanced permeability and retention
FAM	5(6)-carboxyfluorescein
FcγR	Fcγ receptor
GAS	Growth arrest specific
HCC	Hepatocellular carcinoma
HNSCC	Head and neck squamous cell carcinoma
IC	Immunocomplex
IDO	Indoleamine-pyrrole 2,3-dioxygenase
IFN-γ	Interferon gamma
IL-10R	IL-10 receptor
IL-6R	IL-6 receptor
iNOS	Inducible nitric oxide synthase
IRS	Insulin receptor substrate
ITAM	Immunoreceptor tyrosine-based activation motif
JAK	Janus kinase
LIF	leukemia inhibitory factor
M-CSF	Macrophage colony stimulating factor
MerTK	Mer tyrosine kinase
miRNA	MicroRNA
MMP	Matrix metalloproteinase
mUNO	Minimal functional motif derived from UNO peptide
PE	Phycoerythrin
PHB1	Prohibitin 1
PITPNM3	Phosphatidylinositol transfer membrane-associated protein
PRR	Pattern recognition receptor
RBC	Red blood cell
SIRPα	Signal regulatory protein-α
SPHK1	Sphingosine kinase 1
STAT6	Signal transducer and activator of transcription 6
TAM	Tumor-associated macrophage
TGF-β	Transforming growth factor-β
TME	Tumor microenvironment
TNF-α	Tumor necrosis factor-α
TRAILR	TNF-related apoptosis-inducing ligand receptors
TSP-1	Thrombospondin-1
VEGF	Vascular endothelial growth factor
